# Activation of the sympathetic nervous system suppresses mouse white adipose tissue hyperplasia through the *β*1 adrenergic receptor

**DOI:** 10.14814/phy2.13645

**Published:** 2018-04-02

**Authors:** Mary K. Schneider, Bingzhong Xue, Hang Shi

**Affiliations:** ^1^ Department of Biology and Center for Obesity Reversal Georgia State University Atlanta Georgia

**Keywords:** Adipocyte progenitor, adipose hyperplasia, preadipocyte, sympathetic nerve

## Abstract

Adipose tissue (AT) expands via both hypertrophy and hyperplasia during the development of obesity. While AT hypertrophy involves the increase in size of existing adipocytes, hyperplasia is the process of creating new adipocytes from the pool of adipocyte precursor cells (APCs), which includes adipocyte progenitor cells and preadipocytes. Prior studies have implicated a role of the sympathetic nervous system (SNS) in regulation of hyperplasia in white adipose tissue (WAT). Here, we aimed to determine the mechanisms underlying SNS regulation of APC proliferation in mouse WAT. Using flow cytometry with antibodies against various cell surface markers, along with an intracellular marker of proliferation (Ki67), we quantitated the percentages and proliferative status of adipocyte progenitor cells and preadipocytes in the stromal vascular fraction (SVF) of WAT. In vivo SNS activation through cold exposure, as well as in vitro adrenergic stimulation via exposure to the canonical SNS neurotransmitter norepinephrine (NE), inhibited preadipocyte proliferation. Pretreatment with propranolol, a *β*1‐ and *β*2‐adrenergic receptor (AR) antagonist, trended toward rescuing the inhibitory effects of NE in primary cell culture. The selective *β*1‐AR agonist dobutamine diminished preadipocyte proliferation both in vivo and in vitro, whereas the selective *β*2‐AR agonist, salbutamol, promoted proliferation in vitro, suggesting that the *β*1‐AR may mediate the inhibitory effect of NE on preadipocyte proliferation. Taken together, we conclude that SNS activation suppresses preadipocyte proliferation via activation of the *β*1 AR in WAT.

## Introduction

Obesity is increasingly prevalent and is associated with severe health complications. The comorbidities of obesity include three of the four most deadly noncommunicable diseases today including cardiovascular disease, diabetes, and cancer (Pi‐Sunyer 1999; Nishida et al. 2010). Obesity is a prolonged state of energy imbalance due to excess caloric intake over energy expenditure (Tataranni and Ravussin 1997; Jensen et al. 2014). The body's most efficient storage depot for these excess calories is white adipose tissue (WAT) and research has shown that decreasing WAT mass can greatly reduce, if not negate, the harmful effects of these disease states (Adams et al. 2007; Rider et al. 2009).

WAT mass reduction is ideally accomplished through the adoption of a well‐balanced, low‐calorie diet in combination with increased activity levels. A significant proportion of individuals find this strategy very difficult to maintain, however. In many cases the obese patient with severe comorbidities is prescribed medication that can hinder weight loss efforts. For example, those with Type 2 Diabetes are often in need of insulin therapy, which promotes weight gain and further compounds the problem (Apovian et al. 2015). Given the challenges and variable outcomes associated with the current recommended approaches such as restrictive diet and pharmacotherapy, and the urgent need to combat obesity, research is warranted to define alternative means by which WAT mass can be effectively decreased. To uncover additional approaches to WAT mass reduction, we must improve upon our understanding of adipose tissue (AT) biology and the mechanisms controlling WAT growth.

WAT mass expands either by hypertrophy or hyperplasia, or a combination of both processes (Hausman et al. 2001). The size of existing fat cells increases in the process known as hypertrophy, in which fat cells respond to a state of excess energy through the uptake of circulating lipids for storage. Hypertrophic WAT growth has physical limitations since the plasma membranes of individual cells can only be stretched so far. Hyperplasia is the adipogenic process in which adipose precursor cells (APCs) undergo mitosis, proliferation and differentiation to increase the total number of fat cells. Hyperplasia offers WAT the opportunity to extend its capacity to even greater lengths than hypertrophy alone (Faust et al. 1978). WAT hypertrophy has been examined much more extensively than has WAT hyperplasia. A potentially powerful therapeutic approach to combating WAT growth lies in understanding the regulation of WAT hyperplasia.

It is believed that mature white adipocytes are terminally differentiated, but APCs are capable of dividing and proliferating (Spalding et al. 2008; Algire et al. 2013). APCs consist of adipocyte progenitors and preadipocytes, with preadipocytes being more mature than adipocyte progenitors. Both adipocyte progenitors and preadipocytes, however, have committed to the adipose lineage and have the ability to fully differentiate into mature adipocytes. They also both have the capacity to undergo several cycles of mitosis (Tang et al. 2003; Berry et al. 2014). It is important to note that, while the majority of the physical space in WAT is taken up by mature adipocytes, the stromal vascular fraction (SVF) of WAT consists of numerous other cell types and these various other cell types outnumber adipocytes. The SVF of WAT includes mesenchymal stem cells, APCs, macrophages, lymphocytes, epithelial, and vascular cells (Eto et al. 2009).

WAT is directly innervated by sympathetic nerves arising from the sympathetic ganglion chain as evidenced by neuronal tract tracing studies (Youngstrom and Bartness 1995). In addition to the well‐defined role that the sympathetic nervous system (SNS) plays in stimulating lipolysis, previous research has implicated the SNS in limiting WAT growth. Through its neurotransmitter norepinephrine (NE), the SNS possibly inhibits preadipocyte proliferation (Jones et al. 1992; Cousin et al. 1993; Foster and Bartness 2006).

The approaches utilized in these previous studies for the detection of preadipocytes had some limitations, however. The mechanism underlying SNS inhibition of preadipocyte proliferation also has yet to be defined. The overall aim of this study is to investigate the role of the SNS in regulation of APC proliferation and the underlying mechanism. In this study, we utilized multicolor flow cytometry analyses, a more accurate approach that can quantitatively distinguish between adipocyte progenitors and preadipocytes in the pool of APCs with specific antibodies against their cell surface markers (Berry et al. 2014; Church et al. 2014). We expand upon this specificity with intracellular staining of the protein Ki67, a marker for cell proliferation (Scholzen and Gerdes 2000; Hui et al. 2015; Fukano et al. 2016). This methodology allows us to ascertain distinct impacts on the proliferative status of adipocyte progenitors and preadipocytes following SNS activation. We also employed an in vitro primary cell culture system to examine the cellular pathways mediating the SNS's inhibitory effect on APC proliferation.

## Materials and Methods

### Animals

C57BL/6J male mice (The Jackson Laboratory, Bar Harbor, ME) were used for all experiments. Mice were acclimated to the vivarium for a minimum of 3 days prior to the start of a cold exposure experiment, and for 3 weeks prior to the start of an experiment conducted in thermoneutral conditions. Mice were aged 5–7 weeks at the start of each experiment. The animal studies were approved by the institutional animal care and use committee of Georgia State University. All animals were housed in temperature‐controlled facilities with a 12‐h light/dark cycle and free access to food and water. Euthanasia was performed via carbon dioxide inhalation in a euthanasia chamber following AVMA guidelines.

### Tissue harvest and stromal vascular fraction isolation

Bilateral inguinal white adipose tissue (IWAT) depots were harvested from mice. The IWAT was processed following the methodology of Hausman et al. (2008). Collagenase type 1 (Worthington Biochemical Corp., Lakewood, NJ; cat. #CLS‐1) prepared in HEPES buffer (final concentrations: 0.1 mol/L HEPES, 0.12 mol/L NaCl, 50 mmol/L KCl, 5 mmol/L D‐glucose, 1.5% bovine serum albumin, 1 mmol/L CaCl2) was used for the tissue digest. Tissue was digested for ~45–60 min in a shaking water bath. A series of filtration and centrifugation steps followed the digest such that the SVF was sufficiently separated from mature adipocytes. The floating adipocyte layer was removed and discarded. After sufficient suspension of the SVF pellet in media, an aliquot was taken and further mixed in trypan blue solution 0.4% (GIBCO Laboratories, Gaithersburg, MD; cat. #15250). SVF cells were counted using the Cellometer Auto 2000 cell viability counter (Nexcelom, Lawrence, MA).

### Flow cytometric analyses and selection of APCs

For cell staining*,* fixable viability stain 700 ((FVS 700) BD Biosciences, San Jose, CA; cat. #564997) was added at a concentration of 1:500 to each tube, followed by vortexing then incubation at RT for 15 min. To each tube, 2 mL of FACS buffer (D‐PBS with 2 mmol/L EDTA and 0.5% bovine serum albumin) was added and cells were centrifuged again for 5 min at 1500 rpm. The supernatants were decanted and the FACS buffer wash step repeated. To each tube, 0.05 mL of FACS buffer was added along with 2 *μ*L of Mouse BD FcBlock (cat. #553142) and cells were incubated for 30 min on a rotator at 4°C. Surface stains in 0.05 mL Brilliant Stain Buffer (cat. #563794) were added next and cells were typically incubated in these stains overnight on a rotator at 4°C. The seven antibodies used to target surface antigens were as follows: BV510 Rat Anti‐Mouse CD24 (cat. #563115), FITC Hamster Anti‐Mouse CD29 (cat. #555005), PE‐Cy7™Rat Anti‐Mouse CD31 (cat. #561410), Alexa Fluor^®^ 647 Rat Anti‐Mouse CD34 (cat. #560233), APC‐Cy7™ Rat Anti‐Mouse CD45 (cat. #557659), BV421 Rat Anti‐Mouse CD140A (cat. #562774) and BV605 Rat Anti‐Mouse Ly‐6A/E (cat. #563288). All surface antibodies and stain buffers were purchased from BD Biosciences. Surface antibodies were used at concentrations of 1:100 with the exception of Anti‐CD31 and Anti‐CD45, which were used at a concentration of 1:200.

Cells were fixed using a working solution of Fix/Perm Buffer then permeabilized with a working solution of Perm/Wash Buffer. Both buffers were provided in BD Pharmingen's Transcription Factor Buffer Set (cat. #562574). PE Anti‐Mouse Ki67 was the intracellular stain used to assess proliferation (BioLegend, San Diego, CA; cat. #652404) at a concentration of 1:200. PE Anti‐Mouse Ki67 was added to the cells in 1 × Perm/Wash Buffer followed by incubation of ~1 h. Samples were washed and prepared for analyses by resuspending in FACS Buffer.

Unstained and single‐stained cells or compensation beads were used to set voltages and calculate compensation. Fluorescence minus‐one (FMO) controls were prepared to confirm accurate compensation and to assist in drawing gates. FMO controls were those in which a sample was stained with all antibodies except for one. A separate FMO control was prepared for each antibody and, in each case, the one antibody that was missing was considered to be the antigen of interest. The FMO control was compared to a sample that had been exposed to all antibodies. As long as compensation had been calculated correctly, there were no positive events appearing for the antigen of interest in the FMO control sample, whereas there were positive events for the antigen of interest in the analysis of cells stained with all antibodies.

For example, the FMO control for CD24 (FMO‐CD24) consisted of cells incubated in all fluorescent compounds (FVS700, FITC‐CD29, PE‐Cy7™‐CD31, Alexa Fluor^®^ 647‐CD34, APC‐Cy7™‐CD45, BV421‐CD140A, BV605‐Ly‐6A/E and PE‐Ki67) with the exception of BV510‐CD24. The antigen of interest for FMO‐CD24 was CD24. Where compensation had been sufficiently calculated, the specific antibody that was missing (here, CD24) only showed negative events in its dot plot. This technique allowed for greater confidence in interpreting positive events as being attributable to the presence of the antigen of interest rather than due to spectral overlap with other fluorophores. FMO controls also assisted in drawing gates to separate positive from negative events and was especially useful in two‐color plots (Church et al. 2014).

Cells were analyzed on a BD LSRFortessa™ flow cytometer, using BD FACSDiva™software.

### Cell culture protocol

Cells were seeded at a density of ~1.3 × 10^4^ cells/cm^2^ in 6‐well tissue culture plates (Corning, Inc., Corning, NY; cat. #353046). The day on which the cells were plated was termed “Day 0”. On Day 0 cells were inoculated in “plating medium”, which consisted of Dulbecco's Modified Eagle Medium (DMEM)/F‐12 media (GIBCO; cat. #11330) supplemented with 10% fetal bovine serum (FBS) and 1% L‐Glutamine/Streptomycin/Penicillin ((antibiotics) Sigma Aldrich, St. Louis, MO; cat. #G6784). Media was removed and replaced with fresh plating medium on day 1. On day 3 the media was replaced with “low serum medium”, which consisted of DMEM/F‐12 without phenol red (GIBCO; cat. #11039) containing 1% antibiotics and 1% FBS media.

The test period was initiated when the cells were ~70% confluent, which typically occurred on day 4, and testing lasted for ~48 h. This period consisted of cells being exposed to test compounds first prepared in appropriate vehicles (water or organic solvent) then diluted in DMEM/F‐12 supplemented with 1% FBS. Test compounds were ligands or blockers of adrenergic receptors (ARs) and included the pan‐AR ligand L‐(‐)‐Norepinephrine (+)‐bitartrate salt monohydrate ((NE) cat. #A9512), propranolol hydrochloride (dual *β*1 and *β*2‐AR antagonist; cat. #P8688), CGP‐20712A (specific *β*1‐AR antagonist; cat. #C231), ICI‐118551‐hydrochloride (specific *β*2‐AR antagonist; cat. #I127), dobutamine hydrochloride (selective *β*1‐AR agonist; cat. #D0676), and salbutamol (selective *β*2‐AR agonist; cat. #S8260). When cells were exposed to a pretreatment with antagonists or inhibitors prior to adding agonists or activators, the pretreatment lasted for ~1 h. All test compounds were purchased from Sigma Aldrich. After the test period concluded, cells were first washed with 1× Dulbecco's phosphate buffered saline ((D‐PBS) Corning; cat. #21‐031‐CV) followed by the addition of Accutase^®^ cell detachment solution (Innovative Cell Technologies, Inc., San Diego, CA; cat. #AT‐104‐60). Cells were left to incubate for dissociation up to 1 hour at room temperature (RT) in Accutase^®^. After gentle mechanical detachment, the contents of each well were aspirated into its own round‐bottom 12 × 75‐mm polystyrene tube (Corning; cat. #352052). Tubes were centrifuged in a swing‐bucket centrifuge at RT for 5 min at 1400 rpm. The Accutase^®^ supernatant was removed and the pellets resuspended in 0.1 mL 1X D‐PBS.

### Statistical analysis

Statistical analyses were performed, using IBM SPSS Statistics software. For comparisons between two independent samples, *t*‐tests were run for parametric data while the Kolmogorov–Smirnov *Z*‐test was run for nonparametric data. Experiments with larger sample sizes were analyzed using one‐way ANOVA and the appropriate post hoc tests. Differences were deemed statistically significant with *P* < 0.05. Data are means and error bars are expressed as ±SEM.

## Results

### Cold exposure inhibits adipose precursor cell populations

**Figure 1:**
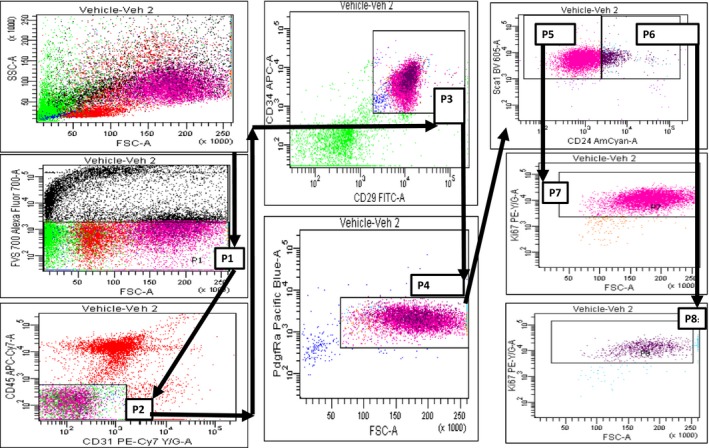
Selection of adipose tissue APCs in a representative flow cytometric analysis. The gate P1 is drawn first to isolate viable cells and sequential gates are created such that only cells included in the previous gate were considered for further selection (i.e., only P1 cells are analyzed when selecting cells for the P2 gate, and so forth); P5 defines preadipocytes while P6 defines adipocyte progenitors; P7 is a dependent of P5, evaluating only preadipocytes for the presence of Ki67; P8 is a dependent of P6, showing only adipocyte progenitors that are positive for Ki67.

Prolonged exposure to cold temperatures is an established stimulator of the SNS (Himms‐Hagen 1967). To test our hypothesis that activation of the SNS inhibits APC populations and proliferation, we exposed mice to the cold (4°C) for 7 days. One group of mice was housed at room temperature (RT) while another was housed in a cold box (4°C). IWAT from each mouse was harvested and processed for flow cytometry analysis. Flow cytometry gating for isolation of APCs is shown in Figure 1. The initial plot of forward scatter (FSC) and side scatter (SSC) was further drilled down to select for live cells. Cells that were negative for FVS700 were considered viable, as necrotic cells allow for the diffusion of FVS700 and binding of the stain to intracellular amines whereas live cells exclude the dye. Cells that did not stain positively for FVS 700 were selected for in the P1 gate. P1 cells were next assessed for CD31 and CD45 expression (endothelial cell and hematopoietic cell markers, respectively) and the gate P2 was drawn around cells that were negative for these markers (CD31‐: CD45‐). P2 cells were further analyzed for enrichment in CD29 (*β*1 integrin) and CD34, a common mesenchymal stem cell (MSC) marker and adipose‐derived MSC (ADMSC) marker, respectively (Calloni et al. 2013; He et al. 2013). The gate P3 was drawn around these cells (CD31‐: CD45‐: CD29+: CD34+). The P3 cell population was then evaluated for the presence of CD140a, commonly known as platelet‐derived growth factor receptor *α* (PdgfR*α*). P3 cells that were positive for PdgfR*α* were captured in the P4 gate (CD31‐: CD45‐: CD29+: CD34+: PdgfR*α*+). The cells in P4 were probed for Ly‐6A/E and CD24 enrichment. Ly‐6A/E is commonly known as stem cell antigen‐1 (Sca‐1) and its expression is indicative of both types of APCs (adipose progenitors and preadipocytes). The divergent factor between adipose progenitors and preadipocytes is the expression of CD24 (also known as Heat‐Stable Antigen). However, adipocyte progenitors are enriched in CD24, preadipocytes lack CD24 expression (Berry et al. 2014; Church et al. 2014). An FMO‐CD24 control was used to draw two gates (P5 and P6) that selected for Sca1+ cells but differentiated between CD24‐ and CD24+ cells in the final two‐color dot plot.

**Figure 2:**
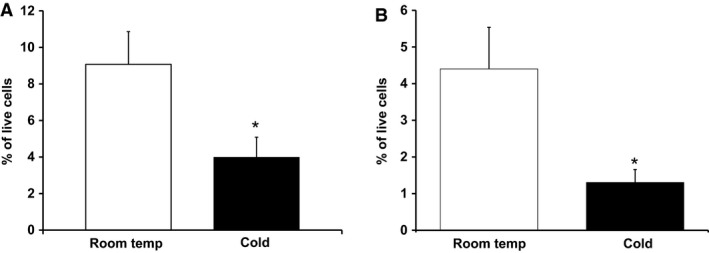
Preadipocyte (A) and adipocyte progenitor (B) cell populations are down‐regulated by cold exposure. Mice were kept at room temperature (*n* = 11) or cold (4‐6 °C) (*n* = 10) for 7 days. Preadipocyte and progenitor cell populations were analyzed by FACS as described in Materials and Methods. Data are expressed as mean ± SEM, *P < 0.05 versus Room Temp.

Preadipocyte population of mice housed at 4°C for 7 days was 4.00%, while RT mice had a significantly greater preadipocyte population of 9.08% (Fig. 2A). Adipocyte progenitor cell populations also were reduced in mice housed at 4°C (1.30% vs. 4.40% in RT mice; Fig. 2B). Taken together, these results suggest that activation of SNS by prolonged cold exposure inhibits both adipocyte progenitor and preadipocyte content in IWAT.

### Norepinephrine inhibits preadipocyte proliferation likely via activation of *β*1‐adrenergic receptors

**Figure 3:**
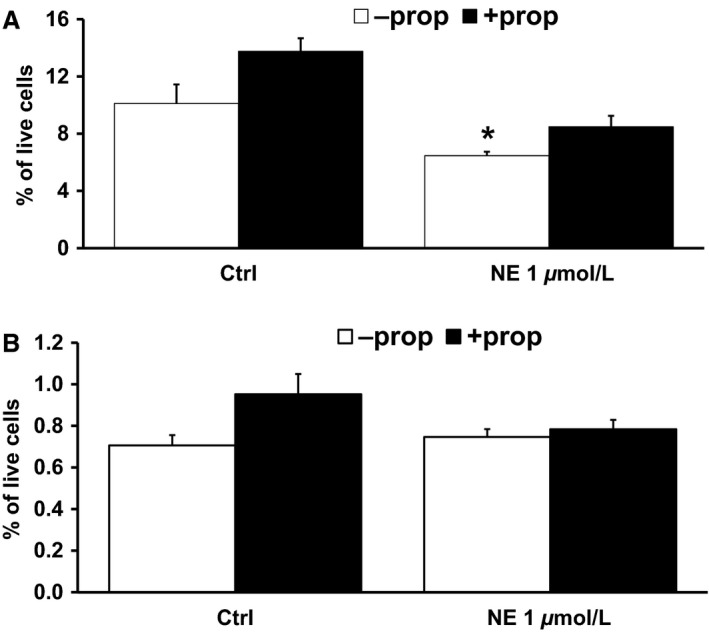
Norepinephrine (NE) decreases preadipocyte proliferation (A) but not adipocyte progenitor proliferation (B). Cells were treated with NE, with or without propranolol (prop) pre‐treatment, for 2 days. Preadipocyte and progenitor cell proliferation were analyzed by FACS as described in Materials and Methods. Data are expressed as mean ± SEM, *n* = 4–6, *P < 0.05 versus control.

Because NE is the principal neurotransmitter released by sympathetic nerves, we next sought to determine the role of NE in APC proliferation. We tested NE's impact, using primary cell culture of mouse IWAT SVF cells. Cells were treated with NE in concentrations ranging from 1 nmol/L to 10 *μ*mol/L for 2 days and the cell proliferation was determined by APC staining coupled with Ki67, using flow cytometry analyses. Cells treated with 1 μM NE had reduced preadipocyte proliferation at 6.47% as compared with controls at 10.11% (Fig. 3A). No effects were seen with other concentrations of NE (data not shown), nor was there any change in adipocyte progenitor proliferation (Fig. 3B). It is of note that NE binds to both *α*‐ and β‐adrenergic receptors (AR), with the *β*‐ARs in particular being expressed on the plasma membranes of murine APCs (Lai et al. 1982; Castan‐Laurell et al. 2002).

To determine which *β*‐AR subtype mediates NE's inhibitory effect, we first tested propranolol, a *β*1‐ and *β*2‐ AR antagonist, because previous research implicates SNS regulation of AT proliferation through these *β*‐ARs in WAT and brown AT (BAT) (Jones et al. 1992; Bronnikov et al. 1999; Lee et al. 2015). Cells pretreated with 5 *μ*mol/L propranolol prior to the addition of 1 *μ*mol/L NE restored preadipocyte proliferation to control levels (Fig. 3A). Propranolol pretreatment of cells exposed to the various other concentrations of NE trended toward increasing preadipocyte proliferation (data not shown). Propranolol did not alter the impact of NE on adipocyte progenitors (Fig. 3B). Blocking the *β*1‐ and *β*2‐ARs seems to rescue the inhibitory effect of NE, implying that NE acts through the *β*1‐ or *β*2‐AR, or both, to hinder preadipocyte proliferation in WAT.

To further distinguish between the *β*1‐ and *β*2‐ARs, we treated cells for 2 days with the selective *β*1‐AR agonist, dobutamine hydrochloride, or the selective *β*2‐AR agonist, salbutamol, in doses ranging from 100 nmol/L to 10 *μ*mol/L.

**Figure 4:**
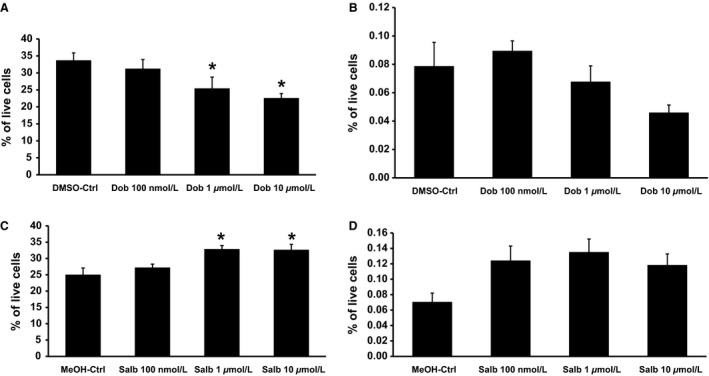
*β*1 adrenergic activation inhibits while *β*2 adrenergic activation promotes preadipocyte proliferations. Treatment of a *β*1 agonist dobutamine (Dob) inhibited preadipocyte proliferation (A) but had no effect on adipocyte progenitor proliferation (B). Treatment of a *β*2 Salbutamol (Salb) stimulated preadipocyte proliferation (C) but had no effect on adipocyte progenitor proliferation (D). Cells were treated with Dob or Salb for 2 days and preadipocyte and progenitor cell proliferation were analyzed by FACS as described in Materials and Methods. Data are expressed as mean ± SEM, *n* = 6–10, *P < 0.05 versus control.

Dobutamine hydrochloride at the concentrations of 1 *μ*mol/L and 10 *μ*mol/L significantly inhibited proliferation of preadipocytes at 25.38% and 22.54% of live cells, respectively, versus controls at 33.66% (Fig. 4A). Adipocyte progenitor proliferation trended downwards but the result was not significant (Fig. 4B).

In contrast, salbutamol at the same concentrations increased preadipocyte proliferation (Fig. 4C). However, proliferating preadipocytes in vehicle‐treated controls accounted for 24.99% of live cells, the percentages of proliferating preadipocytes were increased to 32.81% of cells exposed to 1 *μ*mol/L salbutamol and 32.60% of cells exposed to 10 *μ*mol/L salbutamol. Adipocyte progenitor proliferation trended upwards but was not significant (Fig. 4D). These data support a role of the *β*1‐AR in mediating inhibitory effects on IWAT preadipocyte hyperplasia in vitro. Conversely, activation of the *β*2‐AR appears to promote hyperplasia in vitro.

### The sympathetic nervous system inhibits preadipocyte proliferation through the *β*‐1 adrenergic receptor in vivo

**Figure 5:**
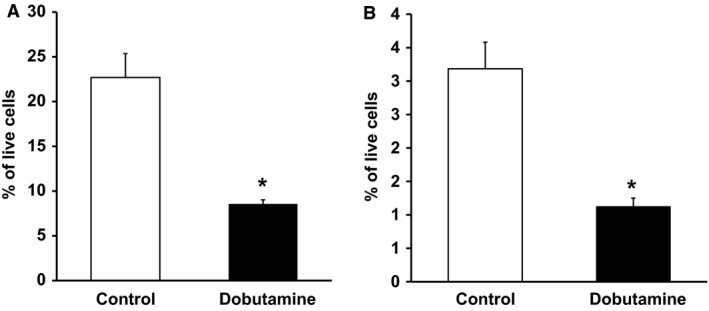
*β*1 adrenergic activation inhibits preadipocyte (A) and adipocyte progenitor (B) proliferation in vivo. Mice housed in thermoneutral conditions were injected with Dob for 7 days. Preadipocyte and progenitor cell proliferation were analyzed by FACS as described in Materials and Methods. Data are expressed as mean ± SEM, *n* = 5–7, *P < 0.05 versus control.

To test the efficacy of dobutamine in inhibiting APC proliferation in vivo, we next administered dobutamine (10 mg/kg) to mice daily via intraperitoneal (ip) injections for 1 week (Fig. 5A–B), with saline ip injections as a control. Because ambient temperature at 22°C can present a mild cold challenge, all animals were housed in thermoneutral conditions (30°C) to minimize the potential inhibitory effect of cold stress on cell proliferation (Cannon and Nedergaard 2011; van der Stelt et al. 2017). We saw significant inhibition of preadipocyte proliferation in the mice injected with dobutamine in comparison with controls (8.57% vs. 22.69% for saline‐injected mice; Fig. 5A). Adipocyte progenitor proliferation was also reduced in dobutamine‐injected mice in comparison to controls (Fig. 5B). The combined in vitro and in vivo data strongly support our assertion that NE released by SNS nerves inhibits WAT preadipocyte hyperplasia via the *β*1‐AR.

## Discussion

Previous studies have examined SNS regulation of WAT hyperplasia, using different techniques to select for APCs from the heterogeneous SVF, but these applications have since been determined to be less accurate and robust in comparison to multicolor flow cytometry. Separation of the adipose fraction via density centrifugation is now known to be vulnerable to contamination with macrophages (Tchoukalova et al. 2012). Histological analysis of WAT sections mandates confident distinction between adipocyte nuclei and nuclei of non‐adipocyte cells. This is very challenging because adipocyte nuclei are localized at the cell's edge alongside the plasma membrane; thus, in the absence of membrane staining, it is difficult to differentiate between adipocytes and other cell types in WAT (Foster and Bartness 2006; Berry et al. 2014). With the more advanced methodology of multicolor flow cytometry, we quantitatively detected the individual adipocyte progenitor and preadipocyte populations and assessed proliferation in the pool of APCs with specific antibodies against their cell surface markers. We found that SNS activation decreased the preadipocyte content and inhibited preadipocyte proliferation in WAT via activation of the *β*1‐AR.

Both adipocyte progenitor and preadipocyte population were diminished in the repeated in vivo cold‐exposure experiments. Activation of the *β*1‐AR via dobutamine in vivo also decreased both adipocyte progenitor cell and preadipocyte proliferation, mimicking the inhibitory effect of SNS activation on APC contents. In contrast, the *β*2‐AR agonist, salbutamol, increased preadipocyte proliferation in vitro. The opposite effect of *β*1‐ and *β*2‐ARs may explain our observation in the in vitro experiment in which NE treatment inhibited preadipocyte proliferation but not that of adipocyte progenitor cells. NE binds to all AR subtypes so it is possible that, in adipocyte progenitors, the *β*1‐and *β*2‐ARs were equally stimulated by NE resulting in a lack of effect on proliferation. We also observed that adipocyte progenitors consistently accounted for a much smaller fraction of APCs as compared to preadipocytes. It is of note that, with preadipocytes being more enriched and further along in the differentiation process than adipocyte progenitors, effects on preadipocytes are perhaps more relevant to our goal of understanding the anti‐obesogenic mechanisms of the SNS. Our data indicate that the *β*1‐AR likely mediates the inhibitory effect of SNS activation on APC contents and proliferation.

Although the relative abundance of *β*1‐AR and *β*2‐AR expression in APC cells is not clear, evidence shows that preadipocytes indeed express *β*1‐AR and *β*2‐AR (Carpene et al. 1983; Guest et al. 1990; Lafontan et al. 1995; Boucher et al. 2002; Monjo et al. 2005). Our studies demonstrate that *β* adrenergic activation by NE mostly inhibits preadipocyte proliferation in WAT while having little to no effect on progenitor cells. The different response to beta adrenergic activation between these two APC cells might be due to a differential expression of *β*1‐AR and *β*2‐AR in respective progenitor cells and preadipocytes, since activation of *β*1‐AR and *β*2‐AR show opposite effects on cell proliferation. It is conceivable that, if the *β*1‐AR is the predominant AR expressed in preadipocytes, activation of *β*1‐ARs would lead to overall inhibition of preadipocyte proliferation.

The role of *α*‐ARs in regulation of APC proliferation is not clear. However, evidence shows that the expression of *α*2‐ARs in rodent fat cell membranes appears to be low in comparison to *β*‐ARs (Carpene et al. 1983; Lafontan et al. 1995; Boucher et al. 2002). That being said, when mice are genetically altered to express the human *α*2A‐AR in mature white adipocytes, and when these *α*2A‐ARs are activated by catecholamines, there are secondary stimulatory effects on proliferation of preadipocytes. This is attributed to the release of a paracrine signal (lysophosphatidic acid (LPA)) from mature adipocytes. LPA interacts with receptors expressed by preadipocytes and downstream effectors ultimately increase proliferative activity (Pages et al. 1999).

Consistent with our findings, Jones et al. (1992), reported that NE inhibits the proliferation of primary cultured rat preadipocytes. When preadipocytes were pretreated with propranolol (the same *β*1‐AR and *β*2‐AR antagonist we used in our research) prior to the addition of NE, NE's effect on proliferation was reversed. Conversely, they pretreated preadipocytes with phenoxybenzamine (*α*1‐AR and *α*2‐AR antagonist) prior to the addition of NE and found that blocking the *α*‐ARs did not reverse the inhibition of preadipocyte proliferation. Their findings supported their conclusion, and lends further weight to our findings, that the SNS inhibits preadipocyte proliferation via one or more of the *β*‐, but not *α*‐, ARs.

Evidence shows that activation of adenosine monophosphate‐activated protein kinase (AMPK) reduces protein synthesis and cell growth, and that AMPK might be a downstream signal of *β*‐AR activation (Moule and Denton 1998; Collins 2011; Almabrouk et al. 2014; Lin et al. 2015). Testing the involvement of AMPK in NE's overall anti‐hyperplasia impact could be a plausible direction to take in elucidating the secondary signaling that follows *β*1‐AR stimulation. It is known that p53 and p21 are proteins that act downstream of AMPK to suppress growth, and these could also be tested with specific inhibitors (Nam et al. 2008; Liu et al. 2013).

The aim of the experiments presented here is to define the role of the SNS in regulation of APC proliferation and the underlying AR mediating the SNS's inhibitory effect. We show that the SNS has significant capacity to limit APC content and growth in WAT, and that it does so via the *β*1‐AR. Putative molecules downstream of *β*1‐AR activation will be tested in future studies. Given the challenges and variable outcomes associated with the current recommended approaches to weight loss, such as restrictive diet and pharmacotherapy, and the urgent need to combat obesity, research is warranted to define alternative means by which WAT mass can be effectively decreased. Understanding the mechanisms by which the SNS suppresses WAT hyperplasia could prove beneficial in our search for additional therapeutic strategies. Additional studies are needed to better understand the downstream signaling of *β*1‐AR activation in the SNS regulation of WAT proliferation.

## Conflict of Interest

None declared.
